# Uncommon Sites of Lymphoepithelial Carcinoma in Head and Neck Region

**DOI:** 10.7759/cureus.36694

**Published:** 2023-03-26

**Authors:** Rathakrishnan Venkatasamy, Viji Ramasamy, Suhana Rahim, Avatar Singh Mohan Singh, Mohd Razif Mohamad Yunus

**Affiliations:** 1 Department of Otolaryngology-Head & Neck Surgery, Hospital Taiping, Taiping, MYS; 2 Department of Otorhinolaryngology-Head & Neck Surgery, Universiti Kebangsaan Malaysia Medical Centre, Kuala Lumpur, MYS; 3 Department of Otorhinolaryngology-Head & Neck Surgery, Hospital Taiping, Taiping, MYS

**Keywords:** base of tongue cancer, parotid malignancy, radiotherapy (rt), head and neck neoplasms, lymphoepithelial carcinoma

## Abstract

We present two cases of Lymphoepithelial carcinoma (LEC) which were found in uncommon sites, the first at the right parotid salivary gland and the second at the base of the tongue. Both patients presented with painless neck masses and were diagnosed with histologic analysis. There is an association with Epstein-Barr virus (EBV) infection in the first case, but none was found in the second case. The primary and metastatic LEC are indistinguishable through histological studies. Therefore, examination of nasopharynx and neck imaging is vital to differentiate primary and metastatic LEC in non-nasopharyngeal sites. A collaboration between surgeons and pathologists is essential for accurate diagnosis of LEC. Radiotherapy is the main choice of treatment for LEC, similar to the cases in the nasopharynx.

## Introduction

Lymphoepithelial carcinoma (LEC) is a distinct type of head and neck tumor classified by the World Health Organization (WHO) [1]. The commonest site of LEC is the nasopharynx and it comprises up to 40% of nasopharyngeal neoplasms [2]. Uncommon cases of LEC have been reported outside the nasopharynx, such as salivary glands, larynx, oral cavity, lip, oropharynx, and others [2-4]. Histologically, nasopharyngeal and non-nasopharyngeal carcinoma have a similar appearance, but the former is associated with Epstein-Barr virus (EBV) [5]. Although LEC is a rare tumor outside the nasopharynx, we have encountered two cases, each originating from the parotid gland and base of the tongue.

## Case presentation

Case 1

A 39-year-old lady with no known comorbidity was referred from a health clinic with one-month history of painless right facial swelling which had shown no noticeable change in size. She had right parotid swelling measuring about 2x2 cm which was not tender. No cervical lymph node was palpable. Her facial nerve was intact. A flexible nasopharyngeal laryngoscope revealed no medialization of lateral pharyngeal wall. Subsequently, fine needle aspiration cytology (FNAC) was taken from the mass and it showed no epithelial cells or atypical cells and was reported as reactive lymphadenopathy. Repeated FNAC revealed atypical cells suspicious of lymphoproliferative disorder, atypical lymphoid cells exhibiting CD20 positivity more than CD3 and negative cytokeratin (CK) AE1/AE3.

**Figure 1:**
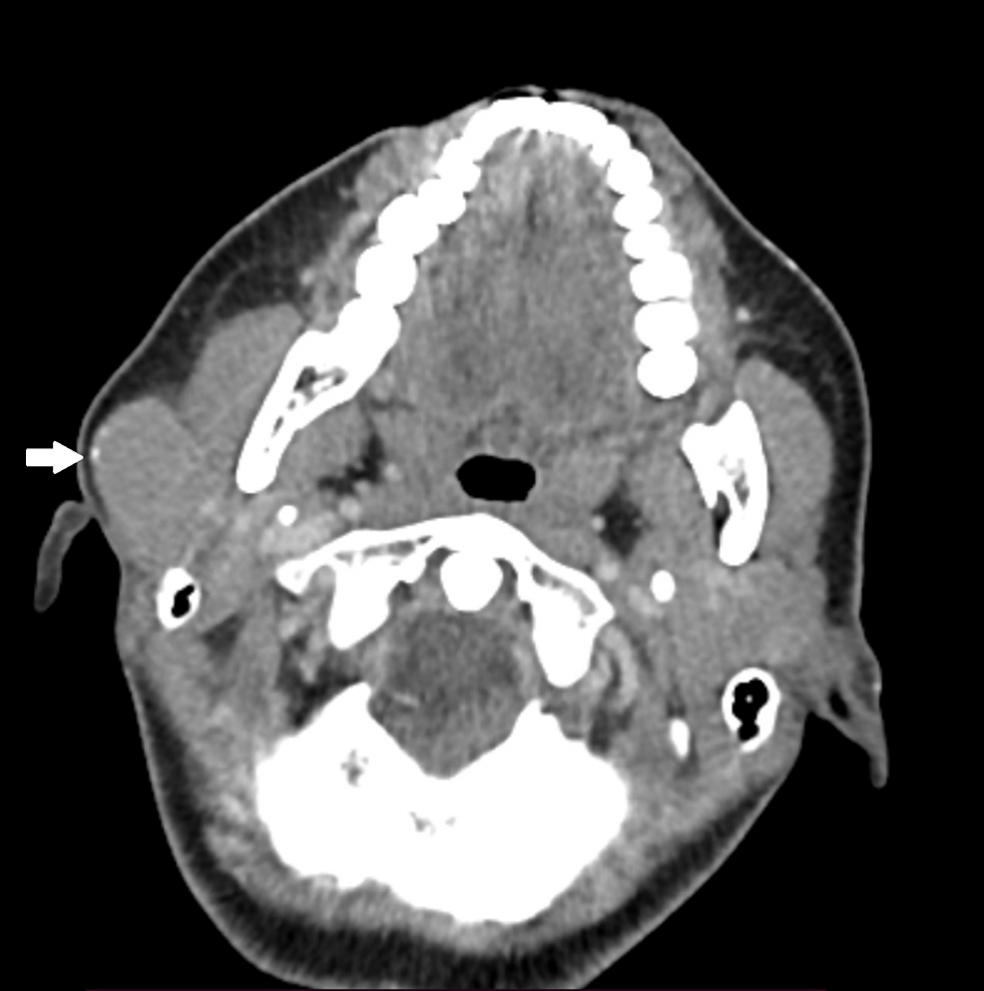
Axial CT image of the parotid gland An axial CT image of the parotid gland demonstrates a homogeneously enhancing lesion at the superficial lobe of the right parotid gland.

**Figure 2:**
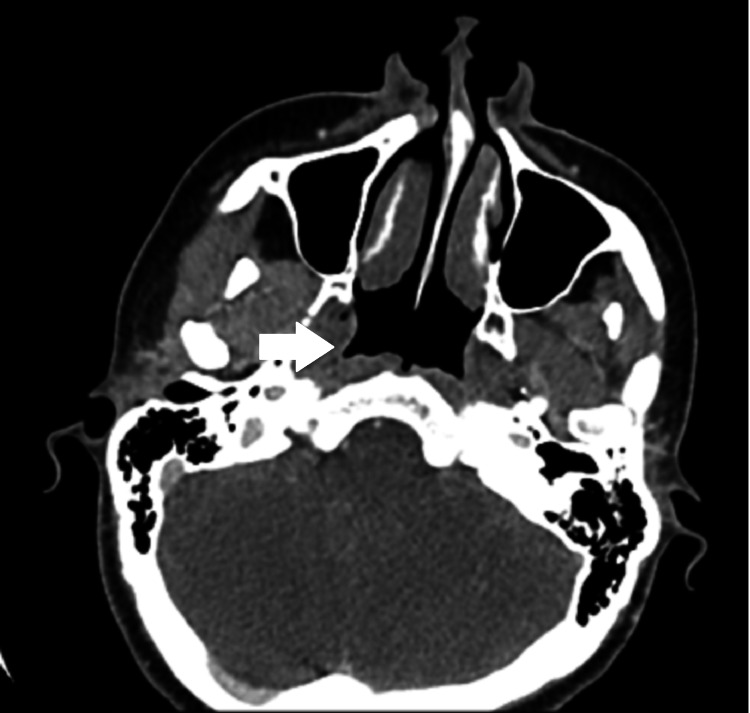
Axial CT image of the nasopharynx An axial CT image of the nasopharynx shows no lesion. The Fossa of Rosenmuller is preserved.

In addition to the FNAC, contrast-enhanced computer tomography (CECT) neck, thorax, abdomen, and pelvis was done. The scan finding was a homogeneously enhancing lesion at the superficial lobe of the right parotid gland approximately 2.1x1.7x2.7 cm in diameter (Figure 1). Fossa of Rosenmuller (Figure 2) was preserved and sub-centimeter lymph nodes at bilateral cervical level II and III were identified. There was no evidence of primary or metastatic lesions in other sites of the neck, thorax abdomen and pelvis.

She underwent a right superficial parotidectomy and a firm mass measuring 3x3 cm was found in the resected gland. All branches of the facial nerve were identified and preserved. The histopathological examination confirmed the diagnosis of LEC. Microscopically, tumor tissue made up of infiltrative sheets and nests of epithelial tumors in a background of intense lymphocytic infiltrate was seen. The tumor was 2 mm from the posterior margin, 3 mm from deep and superior margins, and 5 mm from anterior margin. Immunohistochemistry staining showed positivity towards MNF 116, CKAE1/AE3 and Epstein-Barr virus-encoded small RNA (EBER). Lymphoid components were predominantly CD20 positive. A positron emission tomography (PET) scan found no evidence of local or distant metastasis. After the surgery, patient was referred to an oncology center for radiotherapy which targeted the parotid region and the nasopharynx was not included. She completed her radiotherapy 18 months ago. She had been on regular follow up and no clinical or radiological recurrence was detected for the last two years.

Case 2

A 65-year-old lady with no significant past medical history presented with painless right neck swelling which had no change in size. She had no fever, no nasal blockage, no epistaxis, no dysphagia, no odynophagia and no loss of weight or appetite. On physical examination there was a firm 4x4 cm swelling in the right neck cervical level II, III and IV. No mass was found in the oropharynx. On examination with a flexible nasopharyngolaryngoscope, right base of tongue swelling extending to right lingual surface of the epiglottis obscuring the right side of the vallecula was seen. Bilateral Fossa of Rosenmuller, epiglottis, pyriform fossae, arytenoids and larynx inspection showed no abnormalities. CT scan of neck revealed asymmetry of the hypopharynx with swelling and enhancement over the right side inferior to the vallecula measuring about 1.4 cm. Besides, there was an enlarged and enhanced right upper internal jugular chain lymph node approximately 3x3x4 cm in diameter. Nasopharyngeal recess, parapharyngeal spaces, and all salivary glands appeared normal. Her histopathology report of the right base of the tongue mass biopsy showed undifferentiated carcinoma with no obvious squamous differentiation or keratinization. Immunohistochemistry test identified that the cells are immunoreactive for P40, P63, CK5/6, and CKAE1/AE3. CD3 and CD20 highlighted reactive T and B cell lymphocytes. The tumor cells were tested negative for EBER and p-16. In conclusion, the biopsy was consistent with LEC. Patient was not keen on surgery. Therefore, she was referred to an oncology center for radiotherapy. Patient defaulted our appointment, and surveillance post-radiotherapy was not possible.

## Discussion

LEC is identified by histopathological feature of undifferentiated malignant epithelial cells into lymphoid stroma cells [6]. The most common site of lymphoepithelial carcinoma is the nasopharynx. Tumors with these similar microscopic appearances have been reported in salivary glands, oral cavity, larynx, lips, and others [2-4]. Histologic studies of both cases demonstrated lymphocytes and lymphoblast infiltration in stroma. However, in our first case, initial and repeated FNAC reports were reported as reactive lymphadenopathy and atypical cells suspicious of lymphoproliferative disorder respectively. Second FNAC showed negativity to CKAE1/AE3 test. Cytologic features of LEC are large, single and clustered, polygonal and spindle-shaped tumor cells with a high nucleus-to-cytoplasm ratio in syncytial sheets [7]. Diagnosis of LEC in FNAC is challenging due to its rarity and masking of epithelial elements by a mixed lymphoid population [7]. Histopathological and immunohistochemical examination, mainly AE1/AE3 which highlight cancer cells, is vital in determining a definite diagnosis in the case of lymphoepithelial carcinoma.

In terms of association with EBV, our parotid gland LEC patient was immunoreactive to EBER and the base of tongue LEC patient was negative for EBER. This indicates a positive relation to EBV in our first case and no association with EBV in the base of tongue LEC patient. Nasopharyngeal LEC is heavily linked with EBV. Iezzoni et al reported that there is a consistent association with EBV in salivary gland LEC whereas there is no association in other anatomic sites [8]. Parotid LECs have more than 90% association with EBV [9]. 

The LEC of the parotid gland and base of the tongue commonly manifest as painless neck swelling. Pain, rapid growth of neoplasm and facial nerve involvement increases the possibility of malignancy. However, low-grade malignancies may present atypically [10]. Likewise, both of our patients presented with painless neck swelling and no facial nerve palsy.

Metastatic LEC of nasopharyngeal carcinoma is hardly distinguishable from primary LEC in salivary glands and the base of tongue. Therefore, an endoscopic examination of the nasopharynx and oropharynx is necessary to exclude metastatic disease [11]. Endoscopic examination in our patients showed no abnormalities in nasopharynx and oropharynx. These findings were supported by CT scans to exclude metastatic LEC from nasopharynx.

LEC is widely known as a radiosensitive tumor and the mainstay treatment is radiotherapy (RT) with or without surgery [3]. However, for parotid gland tumors, superficial or total parotidectomy with facial nerve preservation is practiced based on the literature review [12]. For optimal oncologic control postoperative adjuvant radiotherapy is recommended [12]. Our patient with parotid gland LEC underwent right superficial parotidectomy followed by radiotherapy. Similar to the salivary glands, recommended therapy for the base of the tongue LEC is radiotherapy.

## Conclusions

Despite being a rare tumor outside the nasopharynx, cases of primary LEC in various regions of the head and neck have been encountered. In the patients presenting with painless neck swelling with lymph node involvement, LEC, both primary and metastatic, should be considered as part of differential diagnoses. Examination of nasopharynx and neck imaging is vital to differentiate primary and metastatic LEC in non-nasopharyngeal sites. A collaboration between surgeons and pathologists is essential for accurate diagnosis of LEC. Radiotherapy is the main choice of treatment for LEC, similar to the cases in the nasopharynx, and surgery is recommended prior to radiotherapy in LEC of salivary glands.
